# Differential incidence trends of colon and rectal cancers in Hong Kong: an age-period-cohort analysis

**DOI:** 10.1186/s40880-018-0311-2

**Published:** 2018-06-28

**Authors:** Bo Zhang, Shao-Hua Xie, Ignatius Tak-sun Yu

**Affiliations:** 1 Food Safety and Health Research Center, School of Public Health, Southern Medical University, Guangzhou, 510515 Guangdong P.R. China; 2 Department of Preventive Medicine, School of Public Health, Sun Yat-sen University, Guangzhou, 510080 Guangdong P.R. China; 3 Upper Gastrointestinal Surgery, Department of Molecular Medicine and Surgery, Karolinska Institute, Karolinska University Hospital, NS 67, 2nd Floor, 17176 Stockholm, Sweden; 4 JC School of Public Health and Primary Care, The Chinese University of Hong Kong, Hong Kong, SAR China; 5 Hong Kong Occupational and Environmental Health Academy, Hong Kong, SAR China

## Abstract

**Background:**

Colorectal cancer has been the second most common cancer among men and women in Hong Kong since 2012, but the underlying reasons for this increase remain unclear. We describe the incidence trend for colorectal cancer in Hong Kong to explore its etiology within this population.

**Methods:**

The temporal trends in colorectal cancer incidence between 1983 and 2012 were analyzed with joinpoint regressions by sex, age groups, and anatomic sites among adults using data from the Hong Kong Cancer Registry. An age-period-cohort analysis was used to evaluate the effects of age, calendar periods, and birth cohorts on the observed temporal trends.

**Results:**

The incidence of colon cancer among those aged 50 years and older in both sexes increased steadily from 1983 until the mid-1990s and was followed by a slight decrease thereafter, whereas the incidence among those aged 20–49 years decreased steadily from 1983 to 2012. In contrast, the incidence of rectal cancer steadily increased in men and remained stable in women throughout the study period. Significant period and birth cohort effects were observed for colon cancer, whereas period effects on the temporal trends were observed for male rectal cancer.

**Conclusions:**

The incidences of colon and rectal cancers have exhibited divergent patterns between 1983 and 2012 in Hong Kong, indicating heterogeneous etiologies between these two types of cancers. Surveillance of the risk factors related to colon and rectal cancers in the Hong Kong population should be performed, and the increased rectal cancer incidence in males is worthy of extra attention.

## Background

Colorectal cancer (CRC) is a commonly diagnosed cancer in both sexes worldwide [1]. The incidence of CRC varies significantly among countries [2, 3], and nearly 55% of the CRC cases worldwide occur in more-developed countries with Western lifestyles [1]. The incidence of CRC in Asia still ranks among the lowest in the world [2]. However, a number of Asian countries or regions, namely, mainland China, South Korea, Japan, Singapore, and Hong Kong, have experienced such a marked rise in CRC incidence that the CRC incidence in East Asia has surpassed the global average in recent decades [1].

CRC has historically been considered a single type of cancer because the colon and rectum are situated at the end of digestive system and are contiguous in both anatomical position and physiological function. However, it is increasingly being recognized that CRC may not be homogenous in terms of morphological, molecular, and genetic characteristics at different anatomical sites [4]. In addition, notable divergent patterns in the incidence trends of colon and rectal cancers have been observed in many regions around the world [5–7], suggesting etiological heterogeneity of CRC according to anatomical site.

Hong Kong is a unique setting for epidemiological studies of CRC because of the special demographic history of the population, rapid economic development, and social transitions during the past few decades. CRC has been the second most common cancer among men and women since 2012 and is a leading cause of cancer deaths in both sexes in Hong Kong. A total of 4979 (2862 males and 2117 females) newly diagnosed CRC cases occurred in Hong Kong in 2014, with a crude incidence of 68.8 cases per 100,000 individuals; 2034 people died from CRC, which accounted for 28.0% of all cancer deaths in Hong Kong [8].

The temporal trend in CRC incidence in Hong Kong has not been recently updated. To better understand the changing epidemiology of CRC in Hong Kong and the underlying reasons for this change, we analyzed the time trends in the incidence of CRC by anatomic site in Hong Kong over the 30-year period of 1983–2012. We also performed an age-period-cohort analysis to address the effects of age, calendar periods, and birth cohorts on the observed trend. Etiological implications were further considered with reference to possible risk factors. Considering the probable heterogeneous etiologies of colon and rectal cancers, all analyses and interpretations were performed according to these two different anatomical sites.

## Materials and methods

### Data source

All newly diagnosed colon cancer and rectal cancer cases between 1983 and 2012 were identified from the Hong Kong Cancer Registry, a population-based cancer registry with over 95% coverage for most cancers [8]. The Hong Kong Cancer Registry is a member of the International Association of Cancer Registries (IACR) and has contributed regularly to the “Cancer Incidence in Five Continents” series since the 1970s. Mid-year population data for each calendar year were obtained from the Hong Kong Census and Statistics Department.

### Joinpoint regression analysis

Age-standardized annual incidence was calculated by the direct method using the World Health Organization (WHO) 1966 World Standard Population as the reference. Temporal trends in the incidences of colon and rectal cancers were evaluated by sex using the Joinpoint Regression Program (version 4.5.0.0) developed by the US National Cancer Institute (NCI) [9]. The joinpoint regression attempted to identify potential changing points over time in the annual incidence and estimated the annual percent change (APC) in each trend segment under the assumption that the annual rate changed at a constant linear rate on a log scale.

### Age-period-cohort analysis

We further performed age-period-cohort analyses for each gender to examine the effects of age, calendar periods, and birth cohorts on the incidence trends using a newly developed web tool from the US NCI [10]. In brief, age-period-cohort models delineate variance in disease trends over time according to age, period and cohort effects. Age at diagnosis is a surrogate for age-related biological factors and reflects the underlying natural history of the disease. Period effects are usually derived from factors that concurrently affect all age groups. In contrast, cohort effects are attributable to factors that influence specific generations (birth cohorts) and vary in prevalence by generation. A web tool that provided a suite of functions and parameters, including model-based estimators of cross-sectional and longitudinal age-specific rates, period and cohort rate ratios that incorporate the overall annual percent change (net drift), and estimators of the age-specific annual percent change (local drifts), was used for age-period-cohort analysis [10]. Particularly, net drift, which is an analog of the annual percent change in the incidence over the study period, estimates the average annual percentage change in the logarithm of the incidence with adjustment for a non-linear period and cohort effects. Local drift indicates the average annual percentage change in incidence over time across different age groups. Cohort (or period) deviations are orthogonal to the linear trend in the birth cohorts (or calendar periods) and reflect the non-linear cohort (or period) effects incorporated into cohort (or period) rate ratio and local drifts. An important implication of local drifts and/or cohort deviations is that a single-summary, age-standardized rate (ASR) curve and APC value or net drift cannot adequately describe the time trends across age groups. In this study, the incidence data were categorized into 14 5-year age groups (ages 20–24, 25–29, 30–34, 35–39, 40–44, …, 80–84, and ≥ 85 years) and six 5-year calendar periods (1983–1987, 1988–1992, …, and 2008–2012), spanning 19 partially overlapping 10-year birth cohorts indicated by the middle years (1898, 1903, …, and 1988). The calendar period 1993–1997 and the 1938 birth cohort were used as reference categories for estimates of relative risk (RR).

## Results

### Incidence profiles of colon and rectal cancer

In general, the average age-standardized rates between 1983 and 2012 were 23.1 per 100,000 person-years for men and 17.8 per 100,000 person-years for women for colon cancer and 15.8 per 100,000 person-years for men and 9.7 per 100,000 person-years for women for rectal cancer among all age groups. Nearly all cases occurred in individuals 20 years of age or older. A total of 54,984 incident cases of colon cancer and 33,308 cases of rectal cancer were recorded in Hong Kong from 1983 to 2012 for the 20-year + age group, among which nearly 90% of the cases were diagnosed at age 50 years or older, and 10% were diagnosed between the ages of 20 and 49 years.

### Incidence trend of colon cancer

**Fig. 1:**
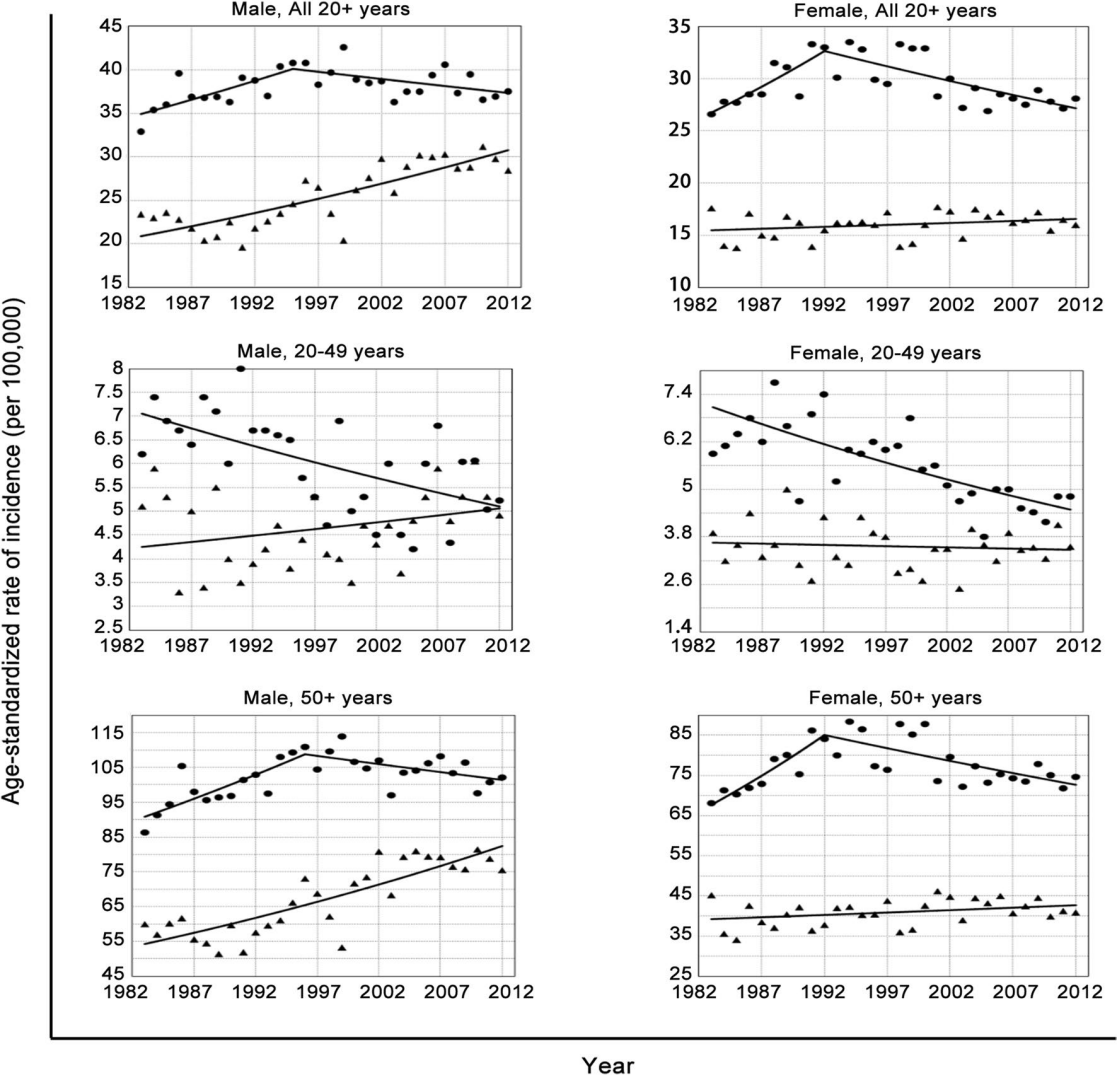
Trends in colon and rectal cancer incidence among men and women by age group, 1983–2012. Rates are age-standardized to the 1966 world standard population. Scattered points are observed rates. Lines are fitted rates according to joinpoint regression, which allowed up to three joinpoints except for male rectal cancer in ages 20–49 years, with 0 joinpoints. Circles = colon cancer; triangles = rectal cancer

Figure 1 shows the age-standardized annual incidence of colon and rectal cancers by sex among individuals in the 20+ age group, 20–49 age group and 50+ age group, and the results from the joinpoint regression of the incidence trends are shown in Table 1. The temporal trends were similar in men and women but differed between the 20–49 and 50+ age groups. Among people aged 50 years or older, the age-standardized incidence of colon cancer in men increased by 1.40% (95% confidence interval [CI] 1.16–1.64%) per year, from 86.3 per 100,000 in 1983 to 110.9 per 100,000 in 1996. This parameter then decreased by 0.44% on average (95% CI − 0.57 to − 0.31%) per year through 2012. Similarly, in women, the age-standardized incidence increased by 2.57% on average (95% CI 2.43–3.11%) per year from 1983 until it peaked in 1992, and then it decreased by 0.78% on average (95% CI − 0.91 to − 0.66%) per year through 2012. In contrast, for individuals in the 20–49 age group, the incidence of colon cancer monotonically decreased in both men (APC = − 1.11%, 95% CI − 1.32 to − 0.90%) and women (APC = − 1.56%, 95% CI − 1.73 to − 1.39%) throughout the entire study period.

**Table 1:** Joinpoint analyses of colon and rectal cancer incidence by sex and age, 1983–2012, Hong Kong

Sex	Anatomic sites	Age	Calendar period	Average APC (95% CI)
Male	Colon cancer	All 20+	1983–1995	1.16 (0.88, 1.44)
			1995–2012	− 0.42 (− 0.54, − 0.30)
			Full Period	0.05 (− 0.02, 0.13)
		20–49	1983–2012	− 1.11 (− 1.32, − 0.90)
		50+	1983–1996	1.40 (1.16, 1.64)
			1996–2012	− 0.44 (− 0.57, − 0.31)
			Full Period	0.21 (0.13, 0.29)
	Rectal cancer	All 20+	1983–2012	1.35 (1.23, 1.46)
		20–49	1983–2012	0.60 (0.37, 0.84)
		50+	1983–2012	1.45 (1.32, 1.58)
Female	Colon cancer	All 20+	1983–1992	2.24 (1.68, 2.79)
			1992–2012	− 0.91 (− 1.04, − 0.79)
			Full period	− 0.33 (− 0.43, − 0.23)
		20–49	1983–2012	− 1.56 (− 1.73, − 1.39)
		50+	1983–1992	2.57 (2.04, 3.11)
			1992–2012	− 0.78 (− 0.91, − 0.66)
			Full period	− 0.12 (− 0.23, − 0.01)
	Rectal cancer	All 20+	1983–2012	0.23 (0.13, 0.33)
		20–49	1983–2012	− 0.17 (− 0.40, 0.05)
		50+	1983–2012	0.29 (0.19, 0.39)

*APC* annual percent change

### Incidence trend of rectal cancer

The temporal trends of rectal cancer differed in men and in women. The age-standardized incidence of rectal cancer in men increased at a steady rate of 1.35% (95% CI 1.23–1.46%) per year over the entire study period. The rate in women fluctuated between a low of 13.8 per 100,000 in 1985 and a high of 17.7 per 100,000 in 2001 and slightly increased over the study period (APC = 0.23%, 95% CI 0.13–0.33%). In both men and women, the rate increased more rapidly in people older than 50 years than in people aged 20–49 years (Table 1 and Fig. 1).

### Age-period-cohort analysis

**Fig. 2:**
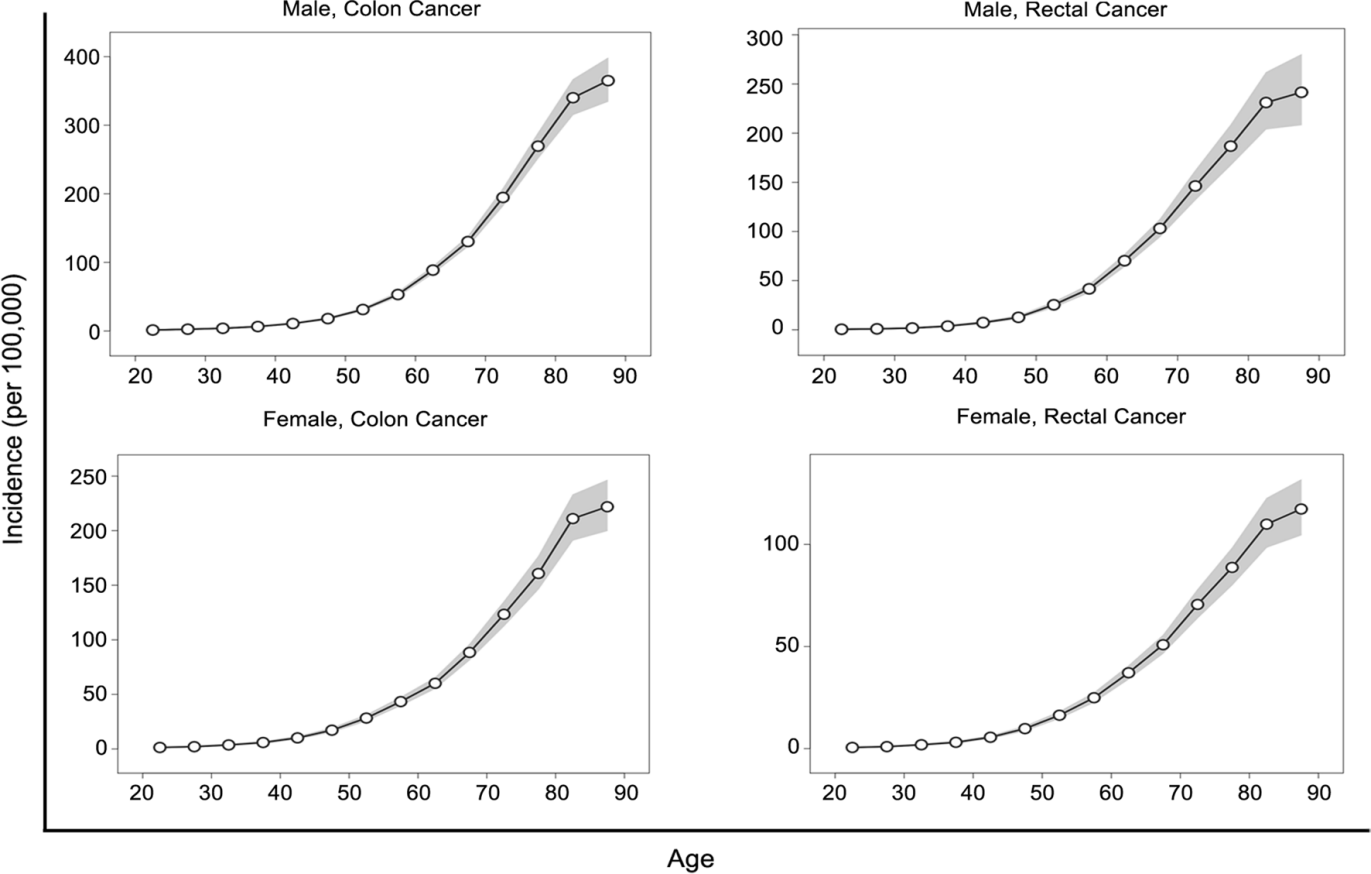
Longitudinal age curves of incidence (1/100,000) for colon cancer and rectal cancer by sex. Shaded regions show pointwise 95% confidence intervals

The fitted longitudinal age-specific incidences of colon cancer and rectal cancer adjusted for period deviations are illustrated in Fig. 2. The longitudinal age curves displayed generally identical patterns across different anatomical sites, indicating a monotonically increased risk with age in both sexes.

**Fig. 3:**
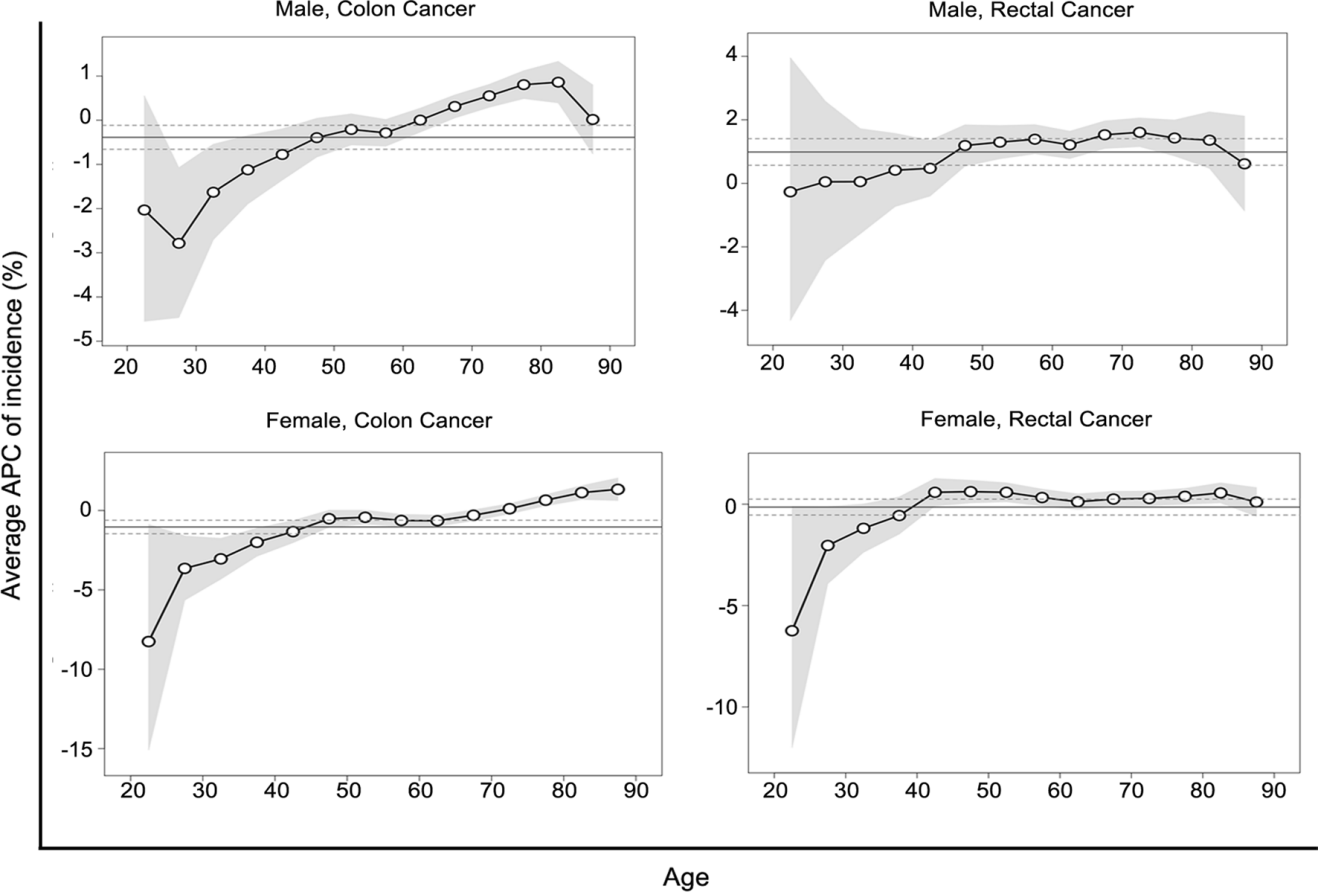
Local drifts for colon and rectal cancer incidence by sex in 1983 and 2012. Shaded regions show pointwise 95% confidence intervals

Local drifts, i.e., age-specific average APCs of the incidences of colon and rectal cancers over time, are illustrated by sex in Fig. 3. The age-specific average APC of colon cancer generally increased with age in both sexes. In contrast, irrespective of sex, no statistically significant differences in the APCs of the incidence of rectal cancer were observed across age groups, although there were seemingly large departures from the net drift for individuals below 40 years of age, with relatively wider 95% CIs compared with individuals over 40 years of age.

**Fig. 4:**
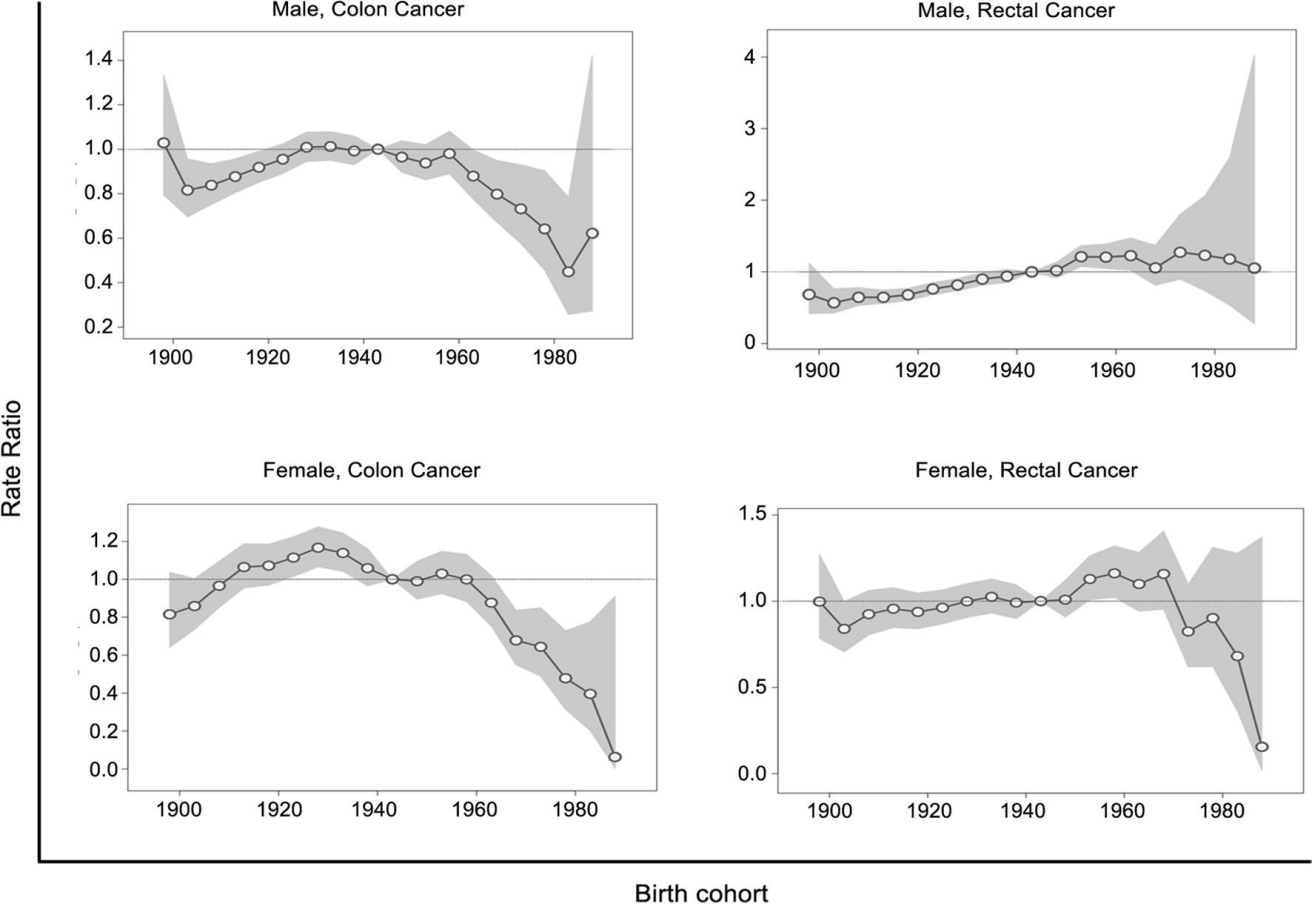
Trends for birth cohort rate ratios of colon and rectal cancer incidence by sex. Shaded regions show the pointwise 95% confidence intervals for rate ratios. The horizontal line is a reference line for rate ratios equal to one

**Fig. 5:**
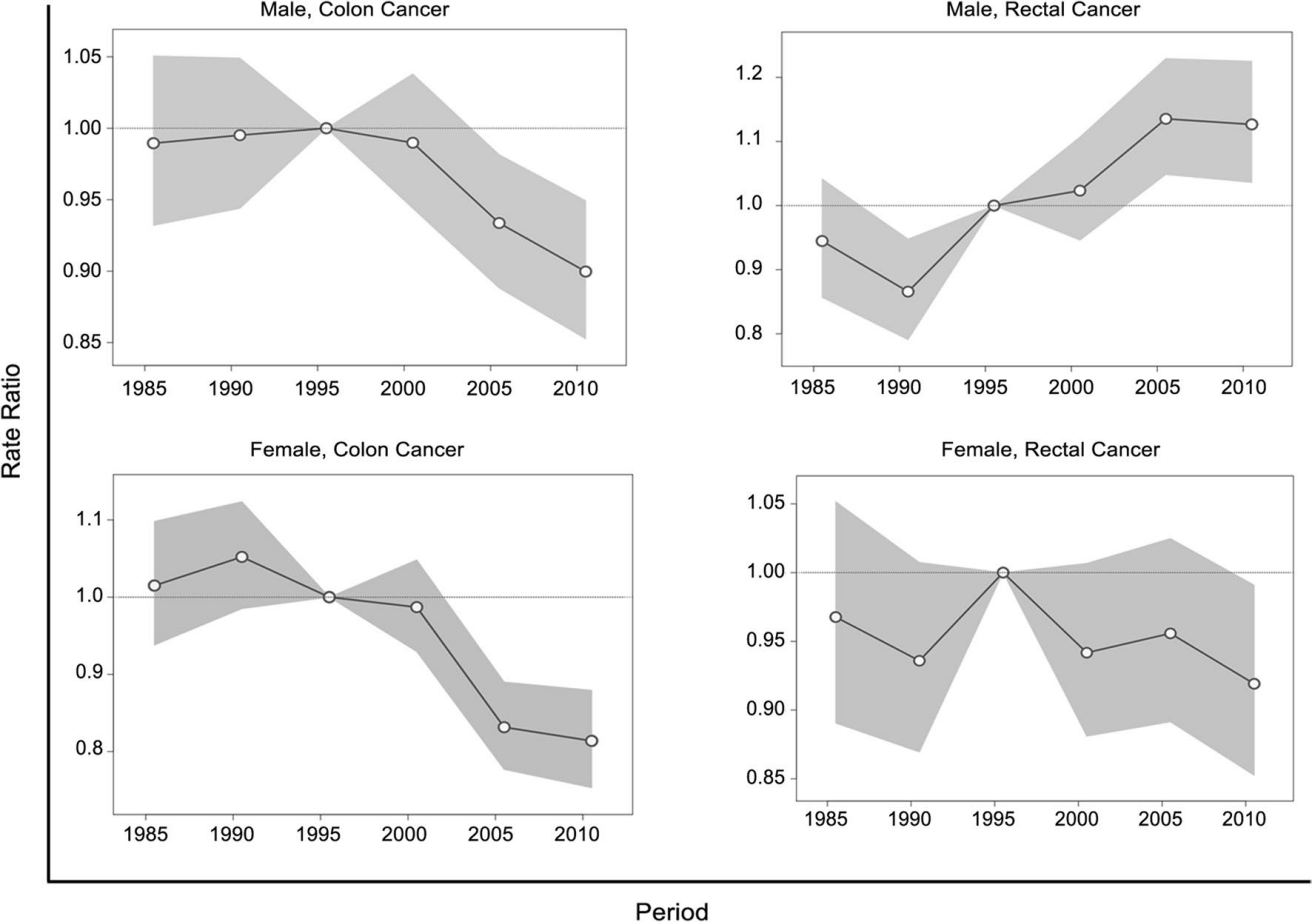
Period effects on colon and rectal cancer incidence by sex. Shaded regions show the pointwise 95% confidence intervals for rate ratios

Figure 4 presents the rate ratios of colon and rectal cancers for each birth cohort by sex. We observed divergent birth cohort effects for colon and rectal cancers in both sexes. The risk of colon cancer in men increased from the early birth cohort until it peaked in the late-1930s cohort, plateaued in the following 15–20 years, and clearly decreased from 1963 forward. Colon cancer risk in women increased from the earliest cohort until the 1928 cohort and decreased steadily thereafter. The risk of rectal cancer generally increased in later birth cohorts for men. For women, the incidence ratio increased slowly until the 1968 birth cohort and decreased abruptly in the later cohorts, but these differences among different birth cohorts showed no statistical significance. The rate ratio plots for calendar periods showed patterns similar to those observed in the joinpoint analyses (Fig. 5).

The Wald test results for the age-period-cohort analyses are presented in Table 2. For the colon cancer incidence trends in both sexes, significant differences in local drifts and cohort deviations indicated potential cohort effects on the observed temporal trends, although period effects could not be ruled out. For rectal cancer, no significant local drifts and cohort deviations in the temporal trends were observed, indicating that there were no cohort effects. The temporal trends in male rectal cancer might be attributable to period effects.

**Table 2:** Statistical parameters for overall and age-specific annual percent changes in age-period-cohort models

Sex	Cancer type	Net drift (% per year; 95% CI)	*P* value		
			All local drifts = net drift	All cohort deviations = 0	All period deviations = 0
Male	Colon	− 0.39 (− 0.66, − 0.12)	< 0.001	< 0.001	0.048
	Rectal	0.99 (0.57, 1.40)	0.562	0.636	0.018
Female	Colon	− 1.04 (− 1.46, − 0.61)	< 0.001	< 0.001	< 0.001
	Rectal	− 0.15 (− 0.54, 0.25)	0.098	0.107	0.210

## Discussion

In this updated analysis of data from the 30-year period between 1983 and 2012, we observed divergent incidence trends for colon and rectal cancers in Hong Kong. Changes in the incidence of colon cancer showed similar patterns in both sexes, with increasing rates until the early 1990s followed by steady decreases thereafter among individuals in the 50+ age group. In addition, a steady decrease in colon cancer incidence between 1983 and 2012 was observed among individuals in the 20–49-year age group. In contrast, the incidence of rectal cancer increased steadily during the entire study period, and this increase was more pronounced in men.

The temporal trends in colon and rectal cancer incidences differ between Hong Kong and Japan, both of which have experienced similar socioeconomic development and were high-income regions in the 1980s. The incidence trends for colorectal cancer in Japan began to differ according to anatomic sites in the early 1990s, when colorectal cancer screening was introduced nationwide [11]. At this time, the incidence of rectal cancer decreased, while that of distal colon cancer stabilized and that of proximal colon cancer continuously increased [11]. Because the Hong Kong government does not offer systematic population-based colorectal cancer screening programs for asymptomatic individuals in any defined age group, we speculated that the observed divergent incidence trends in colon and rectal cancers may be explained by anatomic site-specific environmental factors related to colorectal carcinogenesis. While the possibility of self-initiated screening could not be fully ruled out, it would have had limited influence on the observed incidence trends as it would only have occurred in people with relevant knowledge.

The protective role of dietary factors, such as fruit and vegetable consumption, on CRC has been identified but may be limited to colon cancer only. This is rational to some degree due to the short retention time of fruit and vegetables in the rectum. An earlier systematic review with meta-analysis reported an inverse association between CRC risk and fruit and vegetable intake, which seemed to be restricted to colon cancer only (pooled RR for the highest versus the lowest intake level = 0.91, 95% CI 0.84–0.99) [12]. Another meta-analysis focusing on cruciferous vegetable consumption reported similar results only in terms of an inverse association between cruciferous vegetable intake and CRC risk that was restricted to colon cancer (pooled RR = 0.78, 95% CI 0.69–0.89) rather than rectal cancer (pooled RR = 0.91, 95% CI 0.74–1.13) [13]. Dietary fiber may be one explanation for the inverse association between fresh fruit and vegetable intake and colon cancer risk. Dietary fiber has been hypothesized to reduce the risk of colorectal cancer, although it is unclear whether dietary fiber is independent from other CRC risk factors [14]. A prospective cohort study of Scandinavian populations observed a protective role of total and cereal fiber intake, particularly from cereal foods with a high fiber content, against colon cancer but not rectal cancer; a cereal fiber intake of 2 g per day was associated with a 6% reduced risk of colon cancer in men (incidence ratios = 0.94, 95% CI 0.90–0.98) and a 3% reduced risk in women (incidence ratio = 0.97, 95% CI 0.93–1.00) [15]. A case–control study nested within the European Prospective Investigation into Cancer and Nutrition (EPIC) found an inverse association between plasma levels of alkylresorcinols, biomarkers of whole-grain rye and wheat intake [16, 17], and the risk of distal colon cancer but not rectal cancer [18]. In contrast, the protective role of fish intake against CRC seems more pronounced for rectal cancer (summary OR = 0.79, 95% CI 0.65–0.97) than for colon cancer (summary OR = 0.96, 95% CI 0.81–1.14) [19]. The decreasing age-standardized incidence of colon cancer since the 1990s may reflect the increasing intake of fresh fruit, vegetables and fish in the Hong Kong population [20]. However, this trend is contradicted by the increasing incidence of rectal cancer.

A recent systematic review and meta-analysis reported an increased risk of colon cancer associated with high red meat intake (RR = 1.22, 95% CI 1.06–1.39), but the association for rectal cancer was modestly statistically insignificant (RR = 1.13, 95% CI 0.96–1.34) [21]. How red meat intake influences CRC by anatomical sites and its potential effects on incidence trends for colon and rectal cancers require further research.

The protective effects of physical activity against colorectal cancer also differed by anatomical sites. Physical activity was inversely related to the risk of colon cancer in the proximal (RR = 0.76, 95% CI 0.70–0.83) and distal colon (RR = 0.77, 95% CI 0.71–0.83), but no such relationship could be established for the rectum (RR = 0.98, 95% CI 0.88–1.08) [22]. Due to a lack of surveillance data on physical activity among Hong Kong residents, the contribution of physical activity to the incidence trends of colon and rectal cancers could not be evaluated.

Some risk factors related to colorectal cancer showed a male predominance in either distribution (e.g., smoking and alcohol intake) or the magnitude of the association (e.g., red meat consumption), which might provide some clues to the sex- or anatomical site-specific incidence trends of colorectal cancer. Alcohol use is a well-established risk factor for CRC. A recent meta-analysis revealed a stronger association between heavy alcohol use and CRC risk in Asian populations (pooled RR = 1.81, 95% CI 1.33–2.46) than in Western populations [23], and the elevated CRC risk related to alcohol use does not seem to differ according to anatomical site [24]. A rising trend in alcohol use, in terms of both prevalence and patterns, has been noted in over the past few decades in Hong Kong [25], and this increase parallels the observed increase in the incidence of rectal cancer. However, this observation is not in line with the decreasing incidence of colon cancer over the last 2 decades. It has been suggested that cigarette smoking is associated with an increased risk of both colon cancer (current vs. never smokers, RR = 1.09, 95% CI 1.01–1.18) and rectal cancer (RR = 1.24, 95% CI 1.16–1.39) [26]. Since 1982, the Hong Kong government has taken a progressive approach to tobacco control, raising tobacco taxes and imposing a comprehensive ban in 1999 on tobacco advertising and promotional activities [27]. Therefore, the prevalence of daily cigarette smokers aged 15 years or older decreased from 39.7% in 1982 to 19.1% in 2012 among males [28], which seems to be in line with the decreasing incidence of colon cancer in males over the last two decades. However, we cannot conclude a causal relationship between the decline in the prevalence of cigarette smoking and the decline in the age-standardized incidence of colon cancer because there should be a lag time between these two events, and the prevalence of cigarette smoking before 1980 was scarce. The prevalence of cigarette smoking is low among Hong Kong women, although an increasing trend emerged in 1990 [28]. The diverse temporal trends in the prevalence of cigarette smoking and colorectal cancer suggest that cigarette smoking does not play a major role in the etiology of colorectal cancer among Hong Kong women. The consumption of red meat has been modestly associated with an increased risk of CRC, but the positive association is limited for men. The summary RR estimates for high versus low intake of red meat among men and women were 1.21 (95% CI 1.04–1.42) and 1.01 (95% CI 0.87–1.17), respectively [29]. Therefore, the observed increasing incidence of rectal cancer in men may be, at least to some extent, attributable to the increased intake of red meat in the Hong Kong population [20].

Patients with long-standing inflammatory bowel disease (IBD), i.e., ulcerative colitis and Crohn′s disease, have a 2- to 3-fold increased risk of developing colorectal cancer [30]. Although ulcerative colitis and Crohn’s disease are still relatively rare, their age-adjusted prevalence in Hong Kong, increased from 0.49 to 0.05 per 100,000 in 1985–1989 to 21.14 and 14.17 per 100,000 in 2011–2014, respectively [31]. The dramatic increase in IBD cases in Hong Kong is alarming, however, both the risk factors driving the increase and the long-term consequences of these diseases, such as the risk of colorectal cancer, are unclear.

Diabetes mellitus (DM) is an independent risk factor for colon and rectal cancers with similar magnitudes of association. The association between DM and colon cancer incidence did not differ significantly by sex, but for rectal cancer, there was a significant association between DM and cancer risk for men (summary RR = 1.22, 95% CI 1.07–1.40), but not for women (summary RR = 1.09, 95% CI 0.99–1.19) [32]. The male-specific positive association between DM and rectal cancer is in line with the male-specific increase in the age-standardized incidence of rectal cancer, suggesting that DM may play an important role in the etiology of male rectal cancer in Hong Kong. However, there is a lack of published data on long-term trends in the incidence of diabetes in Hong Kong, and therefore, we cannot confirm a causal relationship.

The present study has some strengths and limitations. The included data from the Hong Kong Cancer Registry were of high quality and had complete coverage. However, because proximal and distal colon cancers were not separately recorded in the Hong Kong Cancer Registry, we were unable to evaluate potential differences in incidence trends despite the increasing concerns regarding the etiological heterogeneity of cancers in these two anatomic subsites. Although we attempted to differentiate between the period and cohort effects on the observed incidence trends, all etiological explanations should be interpreted with caution because of the inherent limitations of age-period-cohort analysis, i.e., the collinearity among age, period, and cohort effects. Intuitively, period effects represent temporal variations in cancer incidence over time that affect all age groups simultaneously, and cohort effects represent differences across groups of individuals born in different eras. In our study, for colon cancer, it was difficult to distinguish between period and cohort effects because they varied in similar ways, while for rectal cancer, it seems that the period effect plays a role. However, the estimated period effects were probably a reflection of cohort effects due to the collinearity between period and cohort effects, instead of “true” period effects.

In summary, the incidences of colon and rectal cancers have changed in divergent patterns in Hong Kong between 1983 and 2012, supporting the belief that these two types of cancers have heterogeneous etiologies. The distinct changes in the epidemiology of colon and rectal cancers cannot be completely explained by current knowledge regarding the etiologies of these two types of cancers. Continuous monitoring of the incidence trends of CRC and research efforts aimed at understanding its etiology by anatomical site are warranted.
